# A Case of Refractory Vernal Keratoconjunctivitis Showing Improvement after the Administration of Upadacitinib for the Treatment of Atopic Dermatitis

**DOI:** 10.3390/diagnostics14121272

**Published:** 2024-06-17

**Authors:** Yoshihito Mima, Eri Tsutsumi, Tsutomu Ohtsuka, Ippei Ebato, Yukihiro Nakata, Taro Kubota, Yuta Norimatsu

**Affiliations:** 1 Department of Dermatology, Tokyo Metropolitan Police Hospital, Tokyo 164-8541, Japan; 2 Department of Ophthalmology, International University of Health and Welfare Hospital, Tochigi 324-8501, Japan; 3 Department of Dermatology, International University of Health and Welfare Hospital, Tochigi 329-2763, Japan; 4 Department of Dermatology, International University of Health and Welfare Narita Hospital, Chiba 286-0124, Japan; norimanorima@gmail.com

**Keywords:** vernal conjunctivitis, atopic dermatitis, T helper 2 cytokine, cyclosporine, dupilumab, upadacitinib

## Abstract

Vernal keratoconjunctivitis is a persistent allergic ocular disease predominantly mediated by the T-helper 2 lymphocyte-associated immune response. The standard therapeutic approaches for vernal keratoconjunctivitis include topical corticosteroids and immunosuppressive eye drops. However, managing vernal keratoconjunctivitis with only topical treatments becomes challenging during seasonally exacerbated periods. Systemic treatments such as oral corticosteroids or cyclosporine may be alternative options. Recently, dupilumab’s efficacy in refractory vernal keratoconjunctivitis treatment has been documented. Here, we report a case of refractory vernal keratoconjunctivitis coexisting with atopic dermatitis that rapidly improved after upadacitinib administration. An 18-year-old Japanese woman presented with atopic dermatitis, vernal keratoconjunctivitis, and hay fever. In winter, the patient experienced widespread erythema and escalated itching, leading to significant discomfort and insomnia. Owing to the difficulty in maintaining her current regimen, upadacitinib (15 mg), a Janus kinase inhibitor was initiated. After upadacitinib administration, the treatment-resistant vernal keratoconjunctivitis and erythema improved. Upadacitinib is beneficial in severe cases of atopic dermatitis. Consequently, in our case, upadacitinib may offer therapeutic benefits for refractory vernal conjunctivitis by improving the T-helper 1/2 type immune response, autoimmunity, and oxidative stress. To our knowledge, this is the first report suggesting the potential utility of upadacitinib in managing severe vernal conjunctivitis.

## 1. Introduction

Vernal keratoconjunctivitis (VKC) is a rare (<1:10,000) chronic allergic condition that affects the surface of the eye. VKC is classified as an ocular allergic disease, representing one of the six recognized subtypes of ocular allergy (along with seasonal allergic conjunctivitis, perennial allergic conjunctivitis, atopic keratoconjunctivitis, contact blepharoconjunctivitis, and giant papillary conjunctivitis). VKC manifests through itching, photophobia, white mucous discharge, lacrimation, the sensation of a foreign body in the eye, and pain associated with corneal shield ulcers. The characteristic signs of VKC include Trantas’ dots, giant cobblestone papillae on the upper eyelids, and shield ulcers [1]. Diagnosis is primarily based on these clinical observations because no definite biomarkers uniquely indicative of the disease have yet been identified [2]. Although VKC typically develops during the first decade of life, it can occur in adults [3]. While VKC usually resolves following puberty, it can result in severe visual dysfunction if inadequately controlled. Patients with VKC progressively develop vision loss, shield ulcers, cataracts, and glaucoma [4].

The underlying pathogenesis of VKC remains unknown [1]. Recently, various studies have hypothesized that both allergies and autoimmune diseases, as well as oxidative stress, contribute significantly to the origin of the disease [5]. Traditionally, VKC was considered to be associated with T helper (Th) 2 cell-mediated immunological processes, which involve the overexpression of interleukin 4 (IL-4), IL-5, IL-9, IL-13, and IL-31, along with the activation of mast cells and eosinophils [6]. VKC is strongly associated with atopic diseases, including atopic dermatitis (AD), characterized by a Th2-shifted immune response [7,8]. Tumor necrosis factor (TNF)-α, one of the Th1 cytokines, plays a crucial role in chronic inflammation, and its levels are elevated on the conjunctival surfaces of VKC [9]. Therefore, not only Th2 cytokines but also Th1 cytokines are implicated in the pathogenesis of VKC. Additionally, autoimmunity has been suggested to play a significant role in the disease’s pathogenesis [10]. Furthermore, oxidative stress is reported to contribute to the persistence of ocular and systemic inflammation alongside the roles of Th1 and Th2 mediators, leading to VKC complications [11]. These findings illustrate the complex interactions between Th1 and Th2 inflammation, autoimmunity, and oxidative stress in the pathogenesis of VKC [9,10,11].

The VKC treatment landscape has various pharmacological options. In mild cases, antihistamine eye drops, mast cell stabilizers, eosinophil inhibition eye drops, and short cycles of topical steroids are the usual treatment options [1]. In moderate-to-severe cases, prolonged corticosteroids and immunomodulatory therapies with cyclosporine or tacrolimus eye drops are strategies to regulate the symptoms [1].

Topical corticosteroid eye drops are effective in managing inflammation through various mechanisms, such as reducing the activity of leukocytes, T helper lymphocytes, and fibroblasts, blocking various cytokine signals and fibroblast proliferation, and interfering with cyclooxygenase 2 activity [12]. However, they should be used for 3–5 days in the acute phase because they have the risk of complications such as cataracts, ocular hypertension, glaucoma, and ocular infections [13,14].

Topical cyclosporine eye drops also have various effects on managing inflammation, including blocking T lymphocyte activation, terminating the production of IL-2 and its receptors, and blocking histamine release from basophils and mast cells [15]. Topical tacrolimus eye drops act on ocular inflammation by blocking IL-2 production, inhibiting IL-3 and IL-4 secretion, and reducing mast cell degranulation [16]. As mentioned above, these topical treatments have been proven effective in managing VKC. In review articles on VKC, the use of topical cyclosporine eye drops is recommended as the first-line therapy, while topical tacrolimus eye drops are considered a second-line option in case the topical cyclosporine treatment fails. It is also recommended that topical corticosteroids should only be reserved for treating acute relapses [5,14].

VKC usually recurs seasonally, especially during the spring and summer [17]. During the seasonally exacerbated period of VKC, it is refractory to these topical treatments, and controlling its condition is challenging [17]. In such cases, oral corticosteroids or cyclosporine are considered alternative options; however, these systemic treatments are generally limited to short-term use because of their side effects [7]. Oral cyclosporine decreases the effect of conjunctival eosinophils and fibroblasts and regulates Th2 cytokines such as IL-4 and IL-13. Thus, cyclosporine improves ocular inflammation and has been approved for refractory VKC treatment [18,19,20]. Moreover, in extremely rare cases, surgical debridement of ulcers, advanced glaucoma, and cataracts is selected [4].

Recently, biological drugs have been used for refractory VKC. Omalizumab, a monoclonal antibody that selectively binds to human IgE, reportedly improved refractory VKC, inhibiting IgE, which underlies the pathogenesis of VKC [21]. Additionally, the effectiveness of dupilumab for refractory VKC treatment has been reported [22]. IL-4 and IL-13 play crucial roles in cellular proliferation and the accumulation of extracellular materials, resulting in giant papillae formation [23]. These giant papillae, along with the inflammatory mediators released by eosinophils and mast cells, can damage the epithelium and lead to shield ulcers and vernal plaques [24]. Therefore, it has been suggested that dupilumab inhibits IL-4 and IL-13 cytokine signaling, resolves giant papillae, and reduces inflammatory mediator overproduction, contributing to the control of refractory VKC [22]. Additionally, it has been documented that tralokinumab, by inhibiting the IL-13 cytokine signal, successfully controlled resistant VKC [25]. Here, we report a case of refractory VKC accompanied by AD that rapidly improved after upadacitinib administration.

## 2. Case Presentation

**Figure 1:**
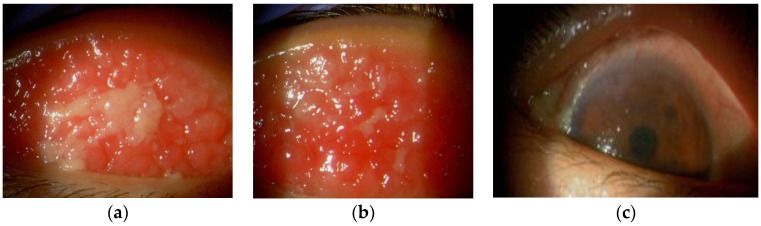
Biomicroscopy revealed giant cobblestone papillae, thickened tarsal conjunctivae, and mucous discharge (right eye: (**a**); left eye: (**b**)). Severe peripheral corneal vascularization, diffuse punctate keratitis, and residual para-central leucomas were also noted (**c**).

An 18-year-old female has presented with AD, VKC, and hay fever since childhood. She had been treated with oral antihistamines and topical steroids for AD and topical cyclosporine and tacrolimus eye drops for VKC. Biomicroscopy revealed giant cobblestone papillae and thickened tarsal conjunctivae, accompanied by a mucous discharge (Figure 1a,b). Additionally, severe peripheral corneal vascularization, diffuse punctate keratitis, and residual paracentral leukomas were observed (Figure 1c).

**Figure 2:**
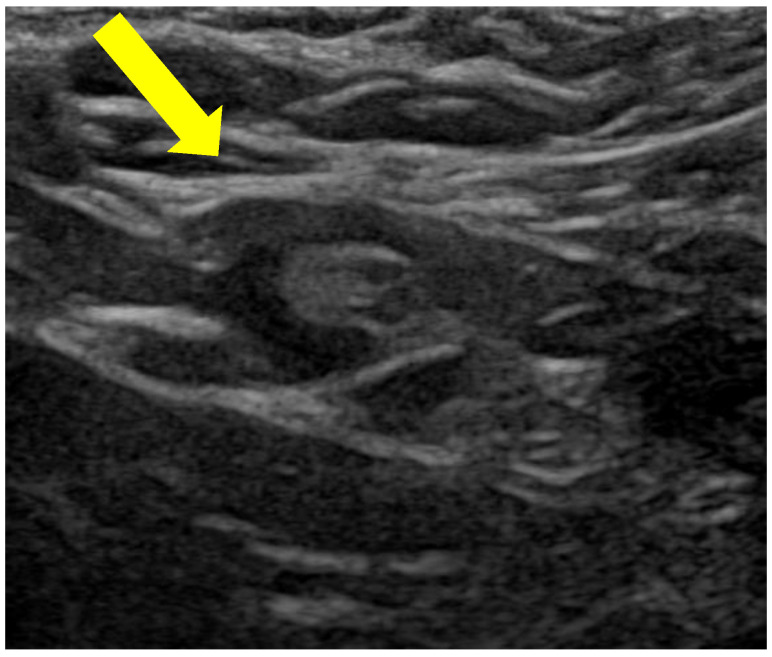
Surface ultrasound echography examination revealed the swollen axillary lymph node, enlarged to a diameter of 15 mm (yellow arrow).

In the winter, she experienced widespread erythema across her entire body and a maximum level of itch score, which significantly impaired her comfort and disrupted her sleep. Moreover, laboratory examinations revealed significantly elevated IgE (8973 IU/mL) and TARC (1602 pg/mL) levels. Surface ultrasonography revealed swollen axillary lymph nodes enlarged to a diameter of 15 mm, indicating a systemic inflammatory condition (Figure 2).

**Figure 3:**
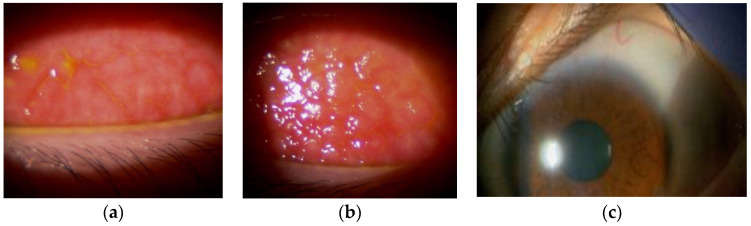
Even in the following spring, giant cobblestone papillae, thickened tarsal conjunctivae, and mucous discharge notably improved (right eye: (**a**); left eye: (**b**)). Severe peripheral corneal vascularization, diffuse punctate keratitis, and residual para central leucomas completely cleared (**c**).

Although we adjusted the strength of the topical corticosteroids and the type of oral antihistamine, the rash did not improve significantly. The severity of her skin condition and itching symptoms caused sleep disturbances, making it difficult for her to continue with the current therapies. Dupilumab and nemolizumab were also considered treatment options, but the patient had an aversion to needles and preferred treatment with oral medication. She desired a fast-acting and potent medication; therefore, we opted for a regimen of upadacitinib, an oral Janus kinase (JAK) inhibitor with highly selective targeting of JAK1. We initiated treatment with a 15 mg dose, considering the possibility of increasing the dosage during exacerbations. Approximately 1 month after upadacitinib administration, severe itching and widespread erythema across her body showed a marked reduction, and the lichenification phase gradually faded. Additionally, the giant papillae and severe peripheral corneal vascularization characteristics of VKC began to flatten within 2 months of upadacitinib initiation, suggesting an improvement in the difficult-to-treat condition of VKC. Even in the following spring, which is typically a challenging period due to pollen allergies, papillary proliferation and peripheral corneal vascularization remained relatively stable (Figure 3a–c).

Five months after starting the administration of upadacitinib, AD and VKC were well managed without any symptoms of discomfort or the occurrence of adverse drug reactions.

## 3. Discussion

VKC is a complex and multifactorial disease characterized by Th2 cell-mediated immunologic processes [6]. Consequently, there is a strong association between VKC and AD, with VKC often occurring concurrently with AD [7,8]. Treatments such as cyclosporine, dupilumab, and tralokinumab inhibit Th2 cytokine signals, including IL-4 and IL-13; thus, these treatments have been approved for severe AD [18,19,20,22,25]. Moreover, they have been reported to effectively control VKC [18,19,20,22,25]. However, no reports have discussed whether upadacitinib is effective for VKC treatment.

The JAK/STAT signaling pathway is crucial for the downstream regulation of various cytokines, playing a vital role in numerous biological, developmental, and homeostatic processes, cell proliferation, and immune regulation. The JAK kinase family consists of four receptor-associated kinases: JAK1, JAK2, JAK3, and TYK2 (tyrosine kinase 2), while the STAT family includes seven proteins: STAT1, STAT2, STAT3, STAT4, STAT5A, STAT5B, and STAT6 [26]. Activated JAKs phosphorylate tyrosine residues on cytokine receptors and other JAKs, creating docking sites that facilitate the recruitment and phosphorylation of key downstream signaling molecules such as STAT proteins [27]. Once phosphorylated, STATs dimerize and translocate to the nucleus, where they bind to DNA and regulate gene transcription. The recruitment of different JAKs and STATs is determined by tissue specificity and the receptors involved in the signaling event [26].

IL-4 binds to type I IL-4R, stimulating the phosphorylation of JAK1 and JAK3, which, in turn, activate and phosphorylate IL-4Rα and STAT6, respectively [28]. IL-4 and IL-13 can bind to type II IL-4R, inducing the robust phosphorylation of JAK1 and TYK2, followed by the activation and phosphorylation of STAT3 and STAT6 [29]. The conjugation of IL-5 and IL-5R triggers the phosphorylation of JAK1 and JAK2, resulting in the activation of STAT1, STAT3, and STAT5 [30]. IL-31 activates JAK1 and JAK2, stimulating STAT3, STAT5, and, to a lesser degree, STAT1 transcriptional activity [31]. The thymic stromal lymphopoietin (TSLP)-mediated response activates STAT5 via the phosphorylation of JAK1 and JAK2 [32]. Thus, JAK1 is instrumental in activating Th2 cytokines, such as IL-4, IL-5, IL-13, and IL-31, and epithelial cell-derived cytokines such as TSLP. Therefore, upadacitinib selectively inhibits JAK1, reducing Th2 and TSLP-driven inflammation. Additionally, IFN-γ, a Th1 cytokine, phosphorylates JAK1 and JAK2 to activate STAT1. As a result, upadacitinib also diminishes Th1 inflammation [33].

Upadacitinib is an oral JAK inhibitor with high selectivity for JAK1. It inhibits the phosphorylation of downstream effector proteins, consequently preventing downstream JAK1-mediated cytokines, including Th2 cytokines [28,29,30,31,32]. Thus, upadacitinib has been approved for treating severe AD [33]. Upadacitinib has been proven to be effective not only for atopic dermatitis but also for rheumatoid arthritis, psoriatic arthritis, ankylosing spondylitis, non-radiographic axial spondyloarthritis, Crohn’s disease, and ulcerative colitis [34].

In the present case, refractory VKC appeared to improve during treatment with upadacitinib for severe AD, along with improvement of the rash. The complex interactions among various factors, such as Th1 and Th2 inflammation, autoimmunity, and oxidative stress, can contribute to the pathogenesis of VKC [9,10,11]. We considered the possibility that upadacitinib inhibits the JAK signaling pathways and significantly improves the overproduction of Th1 and Th2 cytokines involved in VKC pathogenesis, thus ameliorating VKC. Uncontrolled VKC carries the risk of blindness, highlighting the importance of effectively managing this condition [4]. Several cytokines involved in the pathogenesis of autoimmune and inflammatory diseases transduce intracellular signals via the JAK/STAT pathways; therefore, the inhibition of JAK1 leads to the control of autoimmunity [35]. It has been reported that the effect of H_2_O_2_ on ICAM-1 and PD-L1 involves the JAK/STAT pathway; therefore, inhibiting JAK/STAT signaling may ameliorate oxidative stress [36]. Similarly, upadacitinib, by selectively inhibiting JAK1, can improve Th1 and Th2 cytokine production, autoimmunity, and oxidative stress, which are integral to the pathogenesis of VKC. Upadacitinib could potentially be considered a new treatment alternative when conventional therapies fail to control VKC conditions.

To our knowledge, there are no reports on upadacitinib’s effectiveness in VKC treatment. Upadacitinib could be a new treatment option due to its ability to improve Th1 and Th2 cytokine production, autoimmunity, and oxidative stress. Therefore, further accumulation of cases and studies is needed to establish its efficacy.

## Data Availability

Data concerning this article may be requested from the corresponding author for reasonable reasons.
